# Editorial: The HPA axis and aging: individual features, age-related pathology

**DOI:** 10.3389/fendo.2023.1222033

**Published:** 2023-06-02

**Authors:** Nadezhda Goncharova, Douglas Bowden, Elizabeth Johnson

**Affiliations:** ^1^ Laboratory of Experimental Endocrinology, Research Institute of Medical Primatology, Sochi, Russia; ^2^ Department of Psychiatry & Behavioral Sciences and Pharmacology, University of Washington, Seattle, WA, United States; ^3^ School of Medicine, European University Cyprus, Nicosia, Cyprus

**Keywords:** the HPA axis, aging, age-related pathology, individual features, stress

With aging, the frequency of stress-related diseases, including mental, metabolic, cognitive, cardiovascular, and neurodegenerative disorders, increases dramatically. Despite this generalized phenomenon, there are notable individual differences in both vulnerability and resilience to stress and stress-related pathologies. Despite the plethora of studies in stress research, the mechanisms underlying individual vulnerability to stress and age-related diseases remain unclear.

The HPA axis is a key modulator of both the endocrine and behavioral adaptation to stress, and dysfunction of the HPA axis can contribute to the development of various stress-dependent diseases. Function of the HPA axis deteriorates during aging both in basal conditions and in response to stress. However, the type and magnitude of HPA axis deterioration are not similar in all individuals and appear to be associated with different behavioral characteristics. In particular, a higher activity of the HPA axis has been noted in elderly individuals with depression and in non-human primates that exhibit depression- and anxiety-like behavior (DAB) (1–3). Therefore, studying the individual features of the aging HPA axis is important to identify individuals with increased vulnerability to stress and accelerated aging, as is developing a personalized approach for the prevention and treatment of stress-related pathology in the elderly.

Here, we provide a brief editors’ overview of the articles published in this field. The article by Goncharova et al. presents original data on the responses of the HPA axis to acute psycho-emotional stress exposure (ASE) in young and old female rhesus monkeys with standard healthy and DAB behavior under conditions of continuous constant lighting (CL) using LED lamps that are commonly encountered in offices and apartments. The unique findings were that CL reduces the cortisol (CORT) response to ASE applied in the afternoon (15.00-17.00 h) and also disrupts the circadian rhythm of CORT secretion in all animals, regardless of age and behavior.

The mechanisms underlying this phenomenon, however, are age-dependent. In young animals, the transhypophyseal pathway mediates the effect, whereas in older animals the effect is mediated primarily by an extra-hypophyseal neural pathway. The effects of CL on the stress reactivity of the HPA axis in young individuals were similar to its influence on the corticotropin and the CORT responses to administration of arginine vasopressin. A number of individual differences were also noted in DAB animals compared to normal behavioral controls. The findings suggested a potential risk of reduced adaptive capacity under CL resulting in stress-dependent pathology and accelerated aging, especially in the DAB-compromised organism. Such findings are relevant in connection with intensive night lighting and work and other activities at night.

The review by Konstandi and Johnson presents important data on the role of the HPA axis in the regulation of hepatic cytochrome P450 (CYP)-dependent drug metabolism, which is largely genetically determined and not the same in different individuals. Genetic polymorphisms and mutations of CYP genes are observed among several ethnic populations, such as Africans, White and African Americans, Asians, Caucasians, and Europeans. Activation of the HPA axis during stress and aging can lead to disruption of CYP gene regulation and CYP-dependent drug metabolism with pronounced inter-individual variations in the effectiveness and side effects of standard treatment protocols. The implications are particularly important in the treatment of age-related diseases, such as depression, cancer, hypertension, and diabetes. Individual differences in CYP-dependent drug metabolism may also contribute to age-related individual differences in the functioning of the HPA axis.

The article by Degroote et al. focuses on the study of the relationship between individual differences in the daily activity of the HPA axis in middle-aged and elderly people and coronary heart disease (CHD). This is also a main risk factor for arterial hypertension. Both are widespread in these age groups. The authors examined the concentration of CORT in saliva in hypertensive and normotensive men and men with CHD at different times of the day. Additionally, they prospectively tested the cortisol awakening response (CAR) for association with biological risk factors for CHD. They found that lower total daytime CORT secretion and CAR independently predicted increases in cardiovascular risk, as evidenced by increases in the circulating levels of biomarkers of atherothrombotic risk. The findings confirmed the pathogenetic role of age-related disturbances in the HPA axis function in the development of age-related cardiovascular pathology.

One of the main aims of the study presented in Dai et al. was to investigate the role of the HPA axis in the effectiveness of modified electroconvulsive therapy (MECT) for the treatment of drug treatment-resistant depression (TRD). A considerable amount of literature suggests that dysregulation of the HPA axis plays a key role in the pathophysiology of TRD. However, contrary to reports by other authors who identified an association of efficacious electroconvulsive therapy with normalization of the HPA axis dysregulation, these authors found no statistically significant differences in serum CORT levels before and after MECT in elderly patients with TRD. Adequate assessment of the HPA axis function, in this case, may require not just the determination of total blood CORT concentration but also measurement of, for example, dehydroepiandrosterone sulphate and corticotrophin as well. Other confounding factors that may play a role include the time of day when MECT is performed, such as in the evening, a period of reduced circadian HPA axis activity.

Collectively, the articles presented here strongly suggest that the disruption of the HPA axis activity observed across aging has notable deleterious effects on the function of other physiologic systems, namely, 1) altered adaptation to the inhibitory effect of continuous lighting on the cortisol response in acute psycho-emotional stress, 2) modification of the intensity of CYP-dependent drug metabolism, 3) increased vulnerability to cardiovascular pathology, and 4) the daily activity of the HPA axis in basal cortisol concentration and the cortisol awakening response. The general premise supported by most studies is that age-related dysfunctions of the HPA axis form the pathophysiological conditions for the development of age-related pathology and that individual differences in the characteristics and intensity of age-related dysfunctions of the HPA axis may underlie differences in vulnerability to age-related pathology.

## Author contributions

NG wrote the first draft of the manuscript, and DB and EJ revised the manuscript. All authors read and approved the final version.
